# Unrecognised Insanity
**La Folie Lucide:* etudiée et considerée au point de vue de la famille et de la société, par le Docteur Trelat, médecin à l'Hospice de la Salpetrière, &c. Ancien membre du Conseil de Salubrité du Departement de la Seine; Paris, 1861.


**Published:** 1861-10

**Authors:** 


### Art. X.—UNRECOGNISED INSANITY.*

We were consulted some years ago by an unmarried lady of about thirty years of age. She was healthy and robust in aspect, of strong, even masculine intelligence, which had been nurtured and directed by a brother, and her manners were calm and self-possessed. She had moved in a circle containing studious and thinking people, and had busied herself rather with the stern realities than the romance of our lot. She made the following confession. When passing by or near to a window in the street (and the plate-glass era had just commenced), she felt a strong inclination to break the panes: when in church and during sermon, but irrespective of its character and her devotional tendencies—she was pious though not a pietist—she was often impelled to shout, or shriek aloud: and when intrusted with the care of an infant, which frequently happened, she was invariably tempted to crush it, or dash it down upon the floor. This applicant was fully aware that these dispositions were superadded to her natural character; she regarded them as criminal or morbid; she could gaze at and, in a certain sense, speculate upon the impending ruin of her own mind; she concealed, repelled, struggled with, vanquished these impulses; and it was because the violence of her antagonists had increased and because the victory had become doubtful, that she sought medical aid.

Of the category to which this patient belonged M. Trelat treats. His thesis is to describe individuals who are actually insane, whose disease spreads greater misery and misfortune and over a wider sphere than less subtle infirmities, but who are not recognised as labouring under any form of aberration; whose intelligence and cunning are such as to cloak and conceal a substratum of hideous passion or perversity, or to defy and defeat the most probing and penetrating inquiries into their inner life; who combine talents, and energy, and acquirements such as dignify the highest understandings, with gross errors, absurd delusions, and irregularities of conduct; whose reasonable, and clever, and even brilliant conversation gives the lie, nevertheless, to lust, brutality, and degradation; and who come under the Royal definition of "never saying a foolish thing and never doing a wise one."

Such an association must not, however, be confounded with the *Folie raisonante*, for in it the capacity to reason is a part and a characteristic of the malady; in it the form and rules of reasoning are alone preserved, while the premises or conclusions are fal-

---

* *La Folie Lucide:* etudiée et considerée au point de vue de la famille et de la société, par le Docteur Trelat, médecin à l'Hospice de la Salpetrière, &c. Ancien membre du Conseil de Salubrité du Departement de la Seine: Paris, 1861.

lacious; in it a delusion is deftly and dexterously defended, or absurd inferences are logically drawn from inexpugnable data. In the class of cases which M. Trelat has described the disease is apart from, and in despite of, a healthy, or healthlike exercise of judgment; in these cases there run together two straight and unapproachable lines of sane thought and insane emotion or propensity. The treatise might, in fact, be defined—an exposition of forms of moral insanity in which the reasoning powers remain more or less intact. That it enters and occupies that debateable land which embraces the diseases of the Will and of Conscientiousness, and which forms equally the confines of criminality and disease, may be gathered from an analysis of the lucid lunatics who disturb and deceive society, and even the asylums to which they are ultimately consigned.

Among imbeciles and those of feeble intelligence, who are so curtailed and crippled mentally as to be incapable of meeting the dangers and difficulties of life, of discharging their social, family, and private duties; who are disqualified from becoming parents or managing their own affairs; there are individuals susceptible of training, of making vast acquisitions in science and art, who display marvellous constructive, musical, arithmetical talent, and who, under favourable circumstances, differ so little from sane individuals of low grade, as to form a class of enslaved and unpaid labourers in farms and elsewhere: who are denied the benefits, but subjected to the pains and penalties of responsible citizenship. The seduction, the prostitution, the marriage of these imbeciles are recorded. Rational satyrs and nymphomaniacs may be regarded as allied to imbeciles, as being likewise of contracted capacity, although gratifying inordinate appetite while in possession of principles which condemn and could overcome the tendency. Well educated children of twelve and fifteen become prematurely old under the dominion of sexual desires pursued openly, rabidly, in the home of parents. A lady, whose letters indicate great and educated ability and express affection towards her mother, attempts to seduce her father, almost ostentatiously, and, at all events, without a sense of sin or shame, and with a perseverance and ingenuity which savour rather of premeditation than of blind impulse. Wives living on terms of endearment and friendship with their husbands, of sparkling beauty and elegance, surrounded by luxury and splendour, organise "parcs aux cerf," or deliver themselves up to onanism; mothers of fair intelligence prostitute themselves and their daughters, and preserve to the close of life every appearance of propriety of deportment. We have encountered recently two instances in which men reputed sane, and who never otherwise outraged decency, had connexion with their own illegitimate daughters. It may be added that the progeny were idiots. M. Trelat gives the following illustrations of astute monomaniacs, whom he warns us are the most difficult and dangerous class of the insane to deal with: a clever female declares that her head was sawn open by the soldiers of the revolutionary armies, and her moral sentiments extracted, and that she was thus converted into a vile and abject monster, incapable of thinking or doing good: a poor postmistress, compromised by mismanagement, and committed to prison, defends herself at once against the accusation of delinquency and derangement, baffles the repeated and prolonged inquisitions of experts, and composes voluminous expositions of her case, but at last betrays herself by writing in a scarcely visible hand at the close of one of these documents, "I am, we are rich," words which lead to the disclosure, that she believes herself the discoverer of the principles of all things: an educated person devotes herself to serious literary labour, which she pursues ardently, and solitarily, and painfully, but whose studies consist in a calculation how often the letter S, or T, or C, occurs in Genesis, Exodus, Leviticus, the Apocalypse, &c.; how many pages in a particular edition of the Bible commence with D, how many with B, or how many conclude with T, N, or R: an ingenious inventor who ruined his family in attempts to discover perpetual motion, who, disregarding all ordinary motors, conceives that a current of water, nay, even stagnant water, will suffice; and who experimentally, subjected to, and was capable of appreciating the admonitory refutation of Arago, in the presence of Humboldt, whispers, "Arago is wrong, my wheel turns itself." Defining erotomaniacs as instigated by a sentiment in contradistinction to nymphomaniacs, who crave physical gratification, our author adduces an instance of a lover committing suicide on the marriage of one of two sisters, both of whom he had loved with an equally intense attachment; of a lady, who, reasonable in every respect, regulating the affairs of her household, acknowledging the merits of her husband, the tenderness of her relatives, cannot see the one, nor live with the others, because she is devoted to an imaginary lover, who lives in her heart but nowhere else, to whom she is constantly addressing herself and communicating her love by means known only to them.

Such self-deluders live in a state of constant exaltation and tenderness. They write their confessions: they moisten their effusions with tears; they lose sleep, appetite, peace, they court solitude, they subject themselves to suffering and sacrifice as homage to the object of their worship; and yet they may, to the ordinary observer, appear to display, in the transactions of life, sagacity and prudence.

Excessive jealousy is true insanity when it appears in man; he abuses its authority in order to injure, torment, menace, outrage and persecute the object suspected; he may maltreat, wound, murder; when in woman she weeps, cries, becomes excited and violent, and repels and repudiates where she should attract and delight. A sombre, jealous and vindictive mother accuses her husband of violating their youngest daughter; causes his imprisonment, and indirectly the madness of two of their children, but whose horrible suspicion seems to have constituted the only proof of her mental unsoundness.

M. Trelat is desirous of distinguishing drunkards as persons who drink when they have an opportunity of drinking, from dipsomaniacs,who become intoxicated when seized with the paroxysm. It is to be feared that this crux is too simple. In the first place, the morbid craving for stimulants is not always periodic or paroxysmal, but continuous; in the second place, the dipsomaniac creates opportunities for the gratification of his propensity; and in the third place, the phenomena of the tendency may be observed under a great variety of circumstances. It may coexist with the rarest gift of genius, it may be the shadow of the heroic faculties; and, although omnipotent in prostrating and dehumanizing all these attributes of the godlike, be the only speck, the only symptom of disease. It may be the sign of enfeebled or perverted volition. It may be the premonition of mania; the concomitant or consequence of dementia. It may be one of many indications of moral debasement. It may be initiative of optimism in general paralysis; as if the mind potentially foreshadowed its ultimate morbid growth; or sought an immediate realization of the gloomy glories in which imagination is to indulge and expire; or it may be the culmination of a long course of excess and excitement in involuntary and uncontrollable appetite. But although acquired, it may be hereditary; it may appear in childhood, it may be sudden, impulsive. Under all forms it is regarded as all but incurable. The erection of a vast hospital for the treatment of such cases in America, of which, it is averred, thousands desire to become inmates, would indicate that such a discouraging opinion is not universally entertained; and we have grounds for believing that were this propensity recognized and treated as a disease, and were treatment based upon the removal of the physical conditions upon which it so often depends; upon abstinence, and the establishment of new habits, pursuits, and associations, persevered in for a sufficiently long time, it would be found as amenable to such remedies, and as eradicable as other forms of unhealthy impulse. M. Trelat furnishes the familiar example of a youth of promise concluding a complete course of education, which qualified his brothers for excellent appointments, by delivering himself over with fury to the abuse of stimulants, which no advice, no reproach, no prayer, no threat, no authority, no exposure could arrest or alleviate; who converted everything, even his pantaloons, into brandy; upon whom the prison, the asylum, the ministrations of the priest and the physician produced no impression, and who, although he manifested that insensibility to truth, and rectitude and self-respect which is so frequently the characteristic of the confirmed dipsomaniac; after a debauch of twenty years retained unimpaired his recollection, his precision of language, his promptitude in conversation, and many of his acquirements.

Prodigals and adventurers are often clever fools. The motives may be pride, vanity, ambition, but ruin is entailed on the pursuit of gain, and profound policy and selfish purposes may be pleaded in justification of ostentation and profusion. G. is represented as young, fair, highly cultivated, of elevated tone and agreeable manners. She marries, is loved, trusted, and renders her home attractive, both by its decorations and comforts, and by her own manners. It is, however, suddenly discovered that she has indulged in the most lavish expenditure; that, without consideration of her husband's means and situation she has not only spent every shilling, amounting to enormous sums, which was entrusted to her; but that she has involved herself in debt. Carried away by the desire to possess, to buy or expend, she has ordered furniture, dresses, lace, jewellery, which were no sooner in her possession than they were disposed of at a low price, which was again laid out in the purchase of similar and perhaps more extravagant services, stuffs, and gems. In the more advanced stage of the disorder she bought everything, and during one morning secured 2000 francs worth of lace, which she sold for 500, in order to procure a supply of champagne, which was disposed of for a fourth of its price before it reached her cellar. She passed into ambitious mania; her child was idiotic.

Of morbid pride, veiled or relieved by qualities which are accepted as expressions of natural or justifiable principles of action, one illustration may be sufficient. M. N., although young and vigorous, always uses a cab, even in fine weather. He is surrounded by sources of happiness; but his gentle and loving wife, and his children, see little of him, even at the appointed seasons of family reunion. When he invites his friends, he receives them at a restaurant. He has an income of 40,000 francs; but his family enjoy none of the comforts or indulgences of affluence. His means are invested in enterprises such as whale fishing, mining, draining, and the construction of canals. He borrows money from speculators, who boast of doubling or tripling their capital. He is neither manufacturer nor merchant, but opens a credit in various banking houses by profitless transference of his funds. If his customary activity were to relax, were he to give fewer dinners, or squander less money, his credit would suffer. He is therefore the busiest man in the world. In proportion as his speculations fail, or his creditors press, he increases his bustle, and the circulation of his stock. He seeks occasions of profuse expense, in order to maintain and extend his credit. The friends to whom he is indebted conceive that his resources are ten times greater than their own. Ruin merely suggests sacrifices in order to conceal the losses which have taken place. His dissimulation and efforts to conceal his real situation were so successful that, on the eve of his departure, never to return, his credit was good. Even his emigration was dictated, less by the fear of the bankers, than of his friends, whose capital he had employed, not in the pursuit of pleasure, but in order to continue to appear rich. Now, a teacher of languages in a foreign country, his malady is uncured.

There are criminals who do not come within the operation of the legal code. Notwithstanding this immunity, they create greater evil and suffering than the ruffian or the robber. They are conspirators, intriguants against the happiness and wellbeing of society; their arm is against every one; they are false, insincere, mischief-making, but so adroit and expert as to escape the consequences, even the suspicion, of their treachery. The motives for such a course, inexplicable to pure minds, appear in many cases to be merely the triumph of cunning over the innocent and trusting, the disorder and distress which they produce, and the feeling of insecurity which their machinations inspire. Under certain circumstances the disposition they manifest assumes a more practical form. They destroy property in place of confidence, and ruin fortunes as well as reputations; but do so with a superficial sagacity and decorum; and so indirectly as to obtain credit where they merit punishment.

The thieving propensity, which is developed so frequently in extreme youth, and during the most careful judicious culture, is encountered in mature life, in the ripened understanding, and associated with fascinating manners. Kleptomania is generally evinced by the appropriation of articles of no value, in such numbers as to be useless, of a size or nature as to render detection inevitable; but it would be unsafe to infer that the man who steals money and applies it to his own gratification, is therefore insane. He who, in open day, or with a full purse, picks the pocket of his friend, and then bestows the spoil in charity, is as insane as the imbecile who abstracted thirty leagues of black-pudding, and as great a length of sausage as would supply a country. A man of limited intellect, but belonging to an enlightened circle, and who managed his own affairs prudently and profitably; keeps three establishments in Paris; and explains this unusual arrangement by the assertion that he does not relish walking in the streets at night, and that he occupies one or other of these residences, as he may visit in the vicinity. He mingles much in good society, travels, visits watering-places, and wherever the rich and gay most do congregate. His sudden death reveals that each of his apartments are filled, crammed, with sheets, towels, handkerchiefs, vases, umbrellas, pictures, plate, jewellery, &c., which in thirty or forty years he had taken from the houses which he frequented, the friends whose intimacy he had enjoyed. No profit accrued from these thefts; the objects had been turned to no use, and were restored, so far as possible, to their owners. He lived and died unsuspected. A well-educated woman, the mother of a large family, carried on her household so comfortably and elegantly as to excite the astonishment, as well as approval, of her husband. He could not conceive how his limited resources were so judiciously managed as to secure the luxuries by which he and his children were surrounded. Her financial explanation was that by economizing useless and trifling indulgences she could afford to be extravagant; but, after years of impunity, the secret was detected. She was a wholesale plunderer, who had purchased none of those articles which excited the admiration and envy of friends and foes.

It would be superfluous to swell this catalogue by giving examples of the suicidal tendency, coexisting with perfect lucidity; of its being the only symptom of disease; of its forming the sequence of a clear and rigid train of reasoning; or of its being harboured for years, during active and successful pursuits, in minds of apparent strength and integrity, and amidst scenes of mirth and enjoyment. M. Trelat signalizes various curious modes of self-destruction; and in alluding to that by abstinence, states that to Esquirol we owe the great and ingenious idea of conveying nourishment into the stomach, without, and in opposition to the will of the recusant.

It is well known to psychologists, that the most profound stupor, and immobility, and taciturnity may cover faculties of ordinary range, capable of training and exercise. It occurs occasionally that a mere breathing carcase, which lies inert and insensible, which has resisted affection and education, which gives no sign of relation to the external world, of hearing, or seeing, or knowing what is going on around, actually possesses this cognizance; and has been quickened and roused to mental life by a powerful physical impression, such as the shower-bath. Even amongst maniacs there sometimes exists the power of controlling the manifestations of their malady, postponing the paroxysm. For years the world is ignorant of the wild passions which lurk beneath a placid and polite exterior; they engage in business they pay and receive visits, they contract friendships; they may be successful, while their fury is exhausted in solitude, or in the bosom of their family. They may exercise intelligence during the access of excitement; seek the retirement, or adopt the measures which may be required to deceive and protect society; or travel, as was the case where this infirmity was the concomitant of genius, with a strait-jacket in the pocket.

That there are irresponsible and morbid, but intelligent murderers, thieves, evil makers mingled with the sounder elements of society, polluting its purest sources, desecrating the holiest shrines, cannot be denied. That these conspirators against order and harmony are almost, or altogether, madmen is highly probable. But it still remains to be solved—and M. Trelat has not brought us nearer to the solution—whether the mischief and misery caused by such men result from original, indomitable instincts, or so powerful, as to resist all the restraint supplied by the laws and influence of the mind in which they arise, or of the society which they disturb; and whether these instincts be common to all men or characterize the individual, or should be traced to the feebleness of that intelligence which should indicate the consequences, or to the non-existence of that moral sense which should recognise the nature and the relations of the act. There is, unquestionably, disease in each of these conditions, in the inordinate appetite which sets reason and religion at defiance, as well as in the effeteness and insufficiency of conscience and intellect in subduing or extinguishing a passion which is not inordinate. The law, however, as yet admits only intellectual, not moral dementia. Many physicians are too ignorant, or too pusillanimous, to go further. Were it admitted, a very low degree of self-examination would necessitate the admission, that in every act there are involved three distinct elements, the impulse or will, the motive and the perception of effects, and that without the equipoise, and harmony, and fair and full exercise of these principles, no proceeding can be regarded as healthy and spontaneous, the inquiry might be greatly facilitated. Is an act which a man has no purpose in performing, does not intend, or imperfectly intends to perform, and does not understand, his own act? or is not the act a part of his nature? An epileptic gouges his eye out with his own hand, during the irregular and vehement muscular contractions, or exercises of volition, which precede, or take the place of a convulsion; but though the arm was directed and moved by an effort of will, the object was not purposed nor mentally co-ordinated, and the act must be viewed as only partially willed, and as antagonistic to the whole or healthy mind.

How are individuals so influenced to be detected; and intercourse, and social ties and marriage with them, to be avoided?

Those described by M. Trelat, or most of them, were treated as lunatics; but the recognition of their actual condition was generally subsequent to the consummation of calamitous connexions, of marriages entailing wide and ever-spreading wretchedness, and to the public display of some of the more loathsome forms of morbid appetite. As evidence of the magnitude of the evil which he wishes to meet and mitigate, M. Trelat shows that of seventy-seven cases which form the illustrations of his work and in which there remained a certain degree of sanity and self-control, forty-three were demonstrated to belong to tainted races, or to have had members of their family unequivocally insane; while of the same number fifty-one were married and had descendants more or less threatened with a similar fate.

Except in affording most startling proofs of the proposition which he has undertaken to establish, but which had been previously admitted on all hands, and in presenting the carefully prepared results of a long experience, M. Trelat does not appear to have supplied an answer to the question proposed, nor to have suggested other than admonitory expositions of the nature of the process of degeneration going on. He writes, "Qu'on émancipe les forts, qu'on protége les faibles:" but the difficulty consists not in carrying out the noble sentiments, but in the discovery of means by which the feeble, the vitiating and emasculating elements of society are to be recognised and eliminated. The concealment of insanity is itself a symptom of insanity; and even that self-control which subdues the exhibition of passion and perversity, although perhaps deriving influence from the strength created by nervous disease, is so near akin to the highest virtues, the noblest exercise of moral discipline, that, with our existing knowledge, it will be difficult, if not impossible, to determine the exact limits of healthy mental action. M. Trelat seems conscious of the difficulty which must be encountered in admitting the existence of "rational fools," while qualifying his selection of the phraseology employed. He says, "these patients are fools, but do not appear to be fools because they express themselves with clearness. They are fools in their acts, rather than in their words." This attempted definition would include a very large proportion of the inmates of our asylums;—but there is an obvious fallacy, the fallacy of selecting one phenomenon as indicative of health, in calling that individual "perfectly lucid" who merely speaks coherently or rationally, and comports himself decently on certain favourable circumstances; but who when tried and tested, affords ndubitable evidence of insanity.

This treatise must be accepted as a fragmentary chapter in the lifelong experience of the author; as part of a larger work which he seems to despair of completing. It is to be earnestly desired that the apprehension so pensively but naturally expressed in the preface, may not be realized; and that medicine may be still further enriched by the systematized observation of so able and philanthropic a psychologist as the contribution under consideration proves M. Trelat to be. As directing attention to the origin and early history of a class of cases which are rarely met with except in their last stages; as collecting together a large number of examples of the various forms of moral insanity, in which intelligence is preserved, if not intact, unobscured, the present volume is highly valuable.

---

